# Investigation of Energy Band at Atomic-Layer-Deposited ZnO/β-Ga_2_O_3_ ($\overline{2}01$) Heterojunctions

**DOI:** 10.1186/s11671-018-2832-7

**Published:** 2018-12-24

**Authors:** Shun-Ming Sun, Wen-Jun Liu, Yi-Fan Xiao, Ya-Wei Huan, Hao Liu, Shi-Jin Ding, David Wei Zhang

**Affiliations:** State Key Laboratory of ASIC and System, School of Microelectronics, Fudan University, Shanghai, 200433 China

**Keywords:** β-Ga_2_O_3_, Contacts, ZnO, ALD

## Abstract

The energy band alignment of ZnO/β-Ga_2_O_3_ ($\overline{2}01$) heterojunction was characterized by X-ray photoelectron spectroscopy (XPS). The ZnO films were grown by using atomic layer deposition at various temperatures. A type-I band alignment was identified for all the ZnO/β-Ga_2_O_3_ heterojunctions. The conduction (valence) band offset varied from 1.26 (0.20) eV to 1.47 (0.01) eV with the growth temperature increasing from 150 to 250 °C. The increased conduction band offset with temperature is mainly contributed by Zn interstitials in ZnO film. In the meanwhile, the acceptor-type complex defect V_zn_ + OH could account for the reduced valence band offset. These findings will facilitate the design and physical analysis of ZnO/β-Ga_2_O_3_ relevant electronic devices.

## Introduction

Gallium oxide (Ga_2_O_3_) has been widely investigated as a promising ultrawide bandgap semiconductor material for next generation power electronic devices due to its unique properties [1]. Among various polymorphs (α, β, γ, δ, and ε), monoclinic β-Ga_2_O_3_ has the most thermal stability [2]. In addition, β-Ga_2_O_3_ has a room temperature bandgap of 4.5~4.9 eV, and excellent chemical stability [3]. Especially, β-Ga_2_O_3_ has a high bulk electron mobility of ∼100 cm^2^/V·s, much higher breakdown field of 8 MV/cm than that of SiC (3.18 MV/cm) or GaN (3 MV/cm) [4], and the carrier concentration can be easily modulated by doping Sn and Si [5, 6]. Therefore, β-Ga_2_O_3_-based devices including solar-blind photodetectors [7] and metal-oxide-semiconductor field-effect transistors (MOSFETs) [8] have been reported. However, limitations still exist in β-Ga_2_O_3_-based devices, such as the poor ohmic contact between the metal and β-Ga_2_O_3_ [9]. In recent year, inserting a high electron concentration metal-oxide-semiconductor interlayer, i.e., intermediate semiconductor layer (ISL) between the metal and Ga_2_O_3_, has been shown to be an effective resolution because the modulation of energy barrier at the interface [10–12].

Zinc oxide (ZnO) has attracted much attention because it has a large exciton binding energy of 60 meV, a high electron concentration of > 10^19^ cm^−3^, and a strong cohesive energy of 1.89 eV. [13, 14] Additionally, the lattice mismatch between ZnO and Ga_2_O_3_ is within 5% [15]. Various deposition techniques have been developed to prepare ZnO film, including hydrothermal method [16, 17] and chemical vapor deposition (CVD). [18] However, hydrothermal method need a complicated process and the grow rate is quiet slow, and CVD generally requires quiet high growth temperature above 900 °C. These drawbacks make it challenging to be applied in devices. Recently, atomic layer deposition (ALD) has emerged as a promising technique, which exhibits excellent step coverage, atomic scale thickness controllability, good uniformity, and a relatively low deposition temperature. Consequently, atomic-layer-deposited ZnO on wide-bandgap semiconductors can reduce interface disorder and yield more controllable sample to examine the energy band alignment, which plays an important role in the carrier transport process [19]. Up to now, band alignment between Ga_2_O_3_ and atomic-layer-deposited ZnO has not been studied by experiment, although there are some reports about the theoretical band alignment of ZnO and Ga_2_O_3_. [20] Therefore, understanding the energy band alignment of atomic-layer-deposited ZnO/β-Ga_2_O_3_ heterojunction is highly desirable for the design and physical analysis of relevant devices in the future. In this work, the energy band alignment of atomic-layer-deposited ZnO on β-Ga_2_O_3_ was characterized by X-ray photoelectron spectroscopy (XPS). Moreover, the influence of growth temperature of ZnO on the band alignment was also addressed.

## Methods

β-Ga_2_O_3_ ($\overline{2}01$) substrates with a Sn doping concentration of ~ 3 × 10^18^/cm^3^ were diced into small pieces with the size of 6 × 6 mm^2^. The diced samples were alternately cleaned in acetone, isopropanol by ultrasonic cleaning for each 10 min, subsequently rinsed with deionized water to remove residual organic solvents. After that, Ga_2_O_3_ substrates were transferred into an ALD reactor (Wuxi MNT Micro Nanotech co., LTD, China). The growth rate of ZnO films was ~ 1.6 Å/cycle. Both 40 and 5 nm ZnO films were grown on cleaned β-Ga_2_O_3_ using Zn (C_2_H_5_)_2_ (DEZ) and H_2_O at each temperature of 150, 200, and 250 °C, respectively. The thickness of prepared ZnO films was measured by Ellipsometer (Sopra GES-5E). The ZnO(40 nm)/β-Ga_2_O_3_ was used as bulk standard, and the ZnO(5 nm)/β-Ga_2_O_3_ was used to determine the band alignment, in the meanwhile the bare bulk β-Ga_2_O_3_ was used as the control sample. XPS (AXIS Ultra DLD, Shimadzu) measurements with a step of 0.05 eV were performed to measure the valence band maximum (VBM), Ga 2p and Zn 2p spectra. To avoid interference of surface oxidation and contamination, all samples were etched by Ar ion for 3 min with a voltage of 2 kV before XPS measurement. Note that all the XPS spectra were calibrated by C 1s peak at 284.8 eV for compensating the charging effect. To identify the bandgap, the optical transmittance spectra of Ga_2_O_3_ and ZnO were measured by ultraviolet-visible (UV-VIS) spectroscopy (Lambda 750, PerkinElmer, USA).

## Results and Discussion

**Fig. 1:**
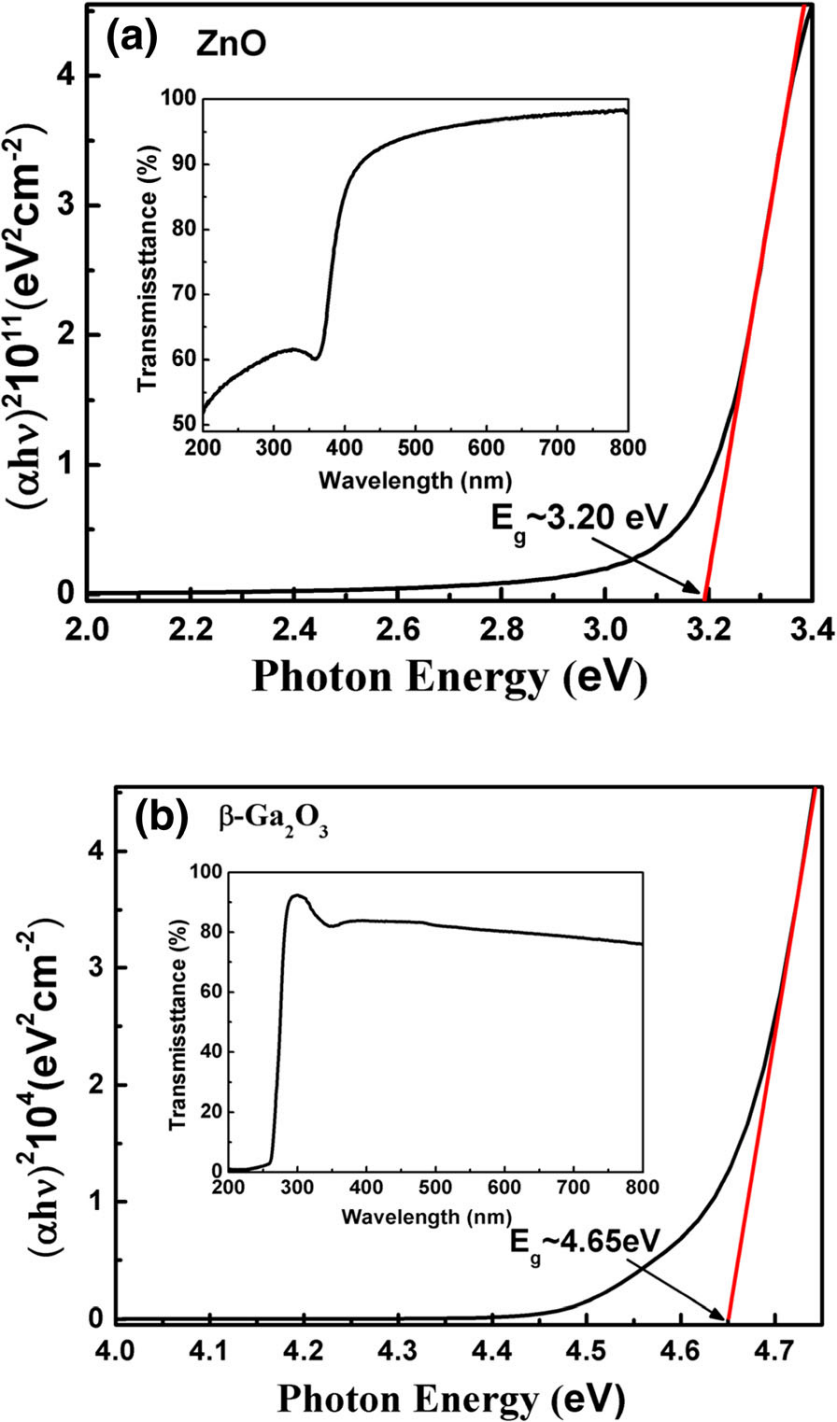
The plot of (α*hv*)^2^ versus *hv* for **a** ZnO film grown on quartz glass **b** β-Ga_2_O_3_ substrate. The inset shows the optical transmission spectra of ZnO and β-Ga_2_O_3_, respectively

Figure 1 shows the variation of (α*hv*)^1/n^ as a function of photon energy for bulk β-Ga_2_O_3_ and the as-grown ZnO film deposited at 200 °C. The optical band gap (*E*_*g*_) of the ZnO film and β-Ga_2_O_3_ can be determined by the Tauc’s relation [21]: (α*hv*)^1/n^ = *A*(*hv* − *E*_*g*_), where α is the absorption coefficient, A is a constant, *hv* is the incident photon energy, *E*_*g*_ is the optical energy bandgap, *n* is 1/2 for the direct bandgap, and 2 for the indirect bandgap. Here, both ZnO and β-Ga_2_O_3_ have typical direct band gap that make the value of *n* is 1/2. Subsequently, *E*_*g*_ can be extracted by extrapolating the straight line portion to the energy bias at α = 0. Therefore, the extracted *E*_*g*_ of ZnO and β-Ga_2_O_3_ are 3.20 eV and 4.65 eV, respectively, in good agreement with the reported. [22, 23]

**Fig. 2:**
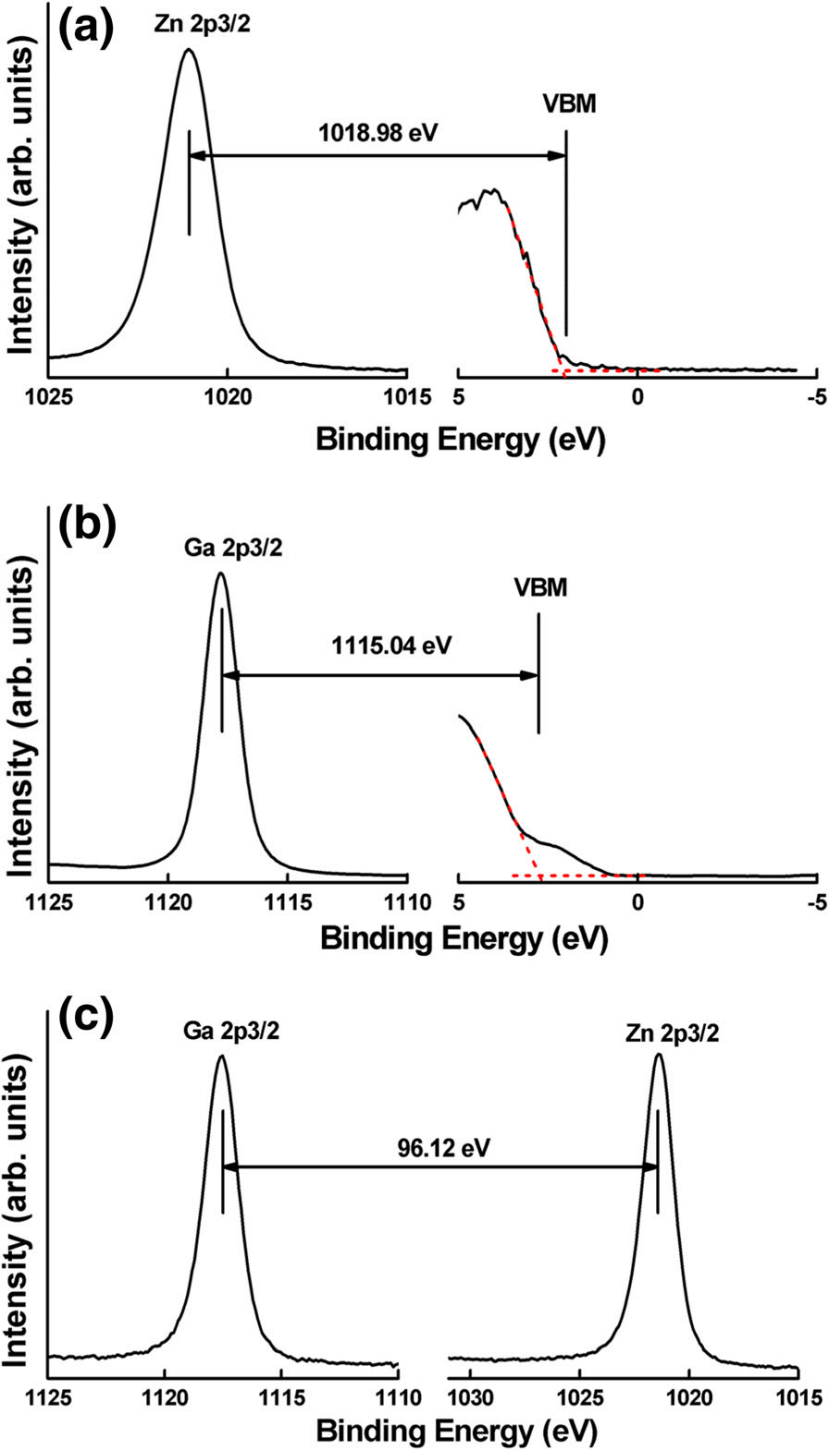
High-resolution XPS spectra for core level and valence band maximum(VBM) of **a** Zn 2p core level spectrum and VBM from 40 nm ZnO/β-Ga_2_O_3_, **b** Ga 2p core level spectrum and VBM from bare β-Ga_2_O_3_, and **c** the core level spectra of Ga 2p and Zn 2p obtained from high-resolution XPS spectra of 5 nm ZnO/β-Ga_2_O_3_

The valence band offset (VBO) can be determined by Kraut’s method using the following formula [24]

1 $$ \Delta  {E}_V=\left({E}_{Ga\ 2p}^{Ga_2{O}_3}-{E}_{VBM}^{Ga_2{O}_3}\right)-\left({E}_{Zn\ 2p}^{Zn O}-{E}_{VBM}^{Zn O}\right)-\left({E}_{Ga\ 2p}^{Ga_2{O}_3}-{E}_{Zn\ 2p}^{Zn O}\right), $$

where ${E}_{Ga\ 2p}^{Ga_2{O}_3}-{E}_{VBM}^{Ga_2{O}_3}$ $\Big({E}_{Zn\ 2p}^{Zn O}-{E}_{VBM}^{Zn O}$) represents to the energy difference between Ga 2p (Zn 2p) core level (CL) and VBM of bulk β-Ga_2_O_3_ (ZnO), and ${E}_{Ga\ 2p}^{Ga_2{O}_3}-{E}_{Zn\ 2p}^{Zn O}$ denotes as the energy difference between Ga 2p and Zn 2p core levels. Figure 2 shows all CL spectra including Zn 2p of ZnO (40 nm)/β-Ga_2_O_3_ and ZnO (5 nm)/β-Ga_2_O_3_, Ga 2p of bulk Ga_2_O_3_ and ZnO (5 nm)/β-Ga_2_O_3_, as well as valence band spectra from bulk Ga_2_O_3_ and ZnO (40 nm)/β-Ga_2_O_3_. Figure 2a presents the CL spectra of Zn 2p on the ZnO (40 nm)/β-Ga_2_O_3_, which is quiet symmetrical indicating the uniform bonding state, and the peak locates at 1021.09 eV corresponds the Zn-O bond [25]. The VBM can be determined using a linear extrapolation method [26]. The VBM of ZnO is located at 2.11 eV. In Fig. 2b, the peak located at 1117.78 eV corresponds to the Ga-O bond [27] and the VBM of Ga_2_O_3_ is deduced to be 2.74 eV according to the method mentioned above. The CLs of Zn 2p and Ga 2p in the ZnO (5 nm)/β-Ga_2_O_3_ are shown in Fig. 2c. According to Eq. (1), the VBO at the interface of ZnO/Ga_2_O_3_ is determined to be 0.06 eV.

**Fig. 3:**
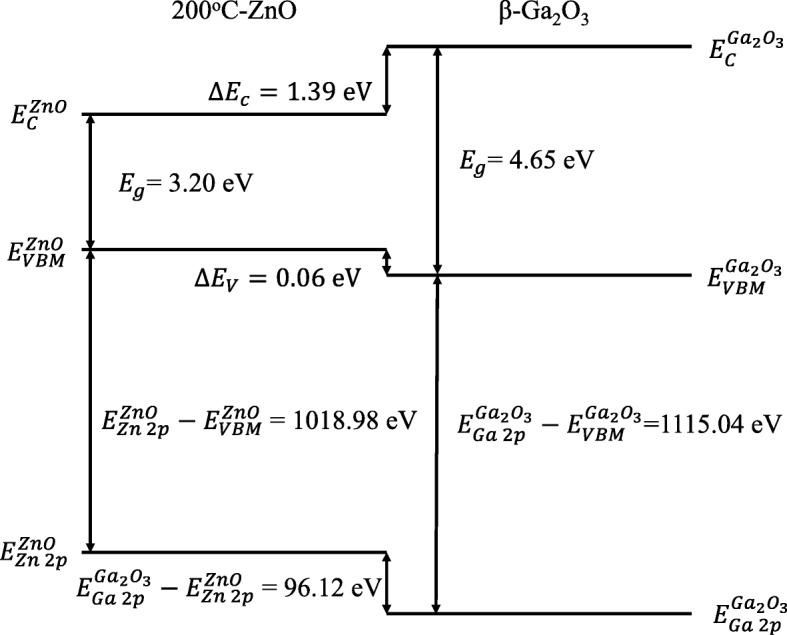
Schematic band alignment diagram of the ZnO (200 °C)/β-Ga_2_O_3_ heterojunction

Based on the calculated *E*_*g*_ and *∆E*_*V*_, the conduction band offset (CBO) at the ZnO/Ga_2_O_3_ interface can be easily deduced from the following equation:

2 $$ \Delta  {E}_C={E}_g^{Ga_2{O}_3}-{E}_g^{ZnO}-\Delta  {E}_V, $$

where${E}_g^{Ga_2{O}_3}$ and ${E}_g^{ZnO}$ are the energy bandgap for β-Ga_2_O_3_ and ZnO, respectively. The detailed energy band diagram for ZnO/β-Ga_2_O_3_ is depicted in Fig. 3. The interface has a type-I band alignment, where both conduction and valence band edges of ZnO are located within the bandgap of β-Ga_2_O_3_.

**Fig. 4:**
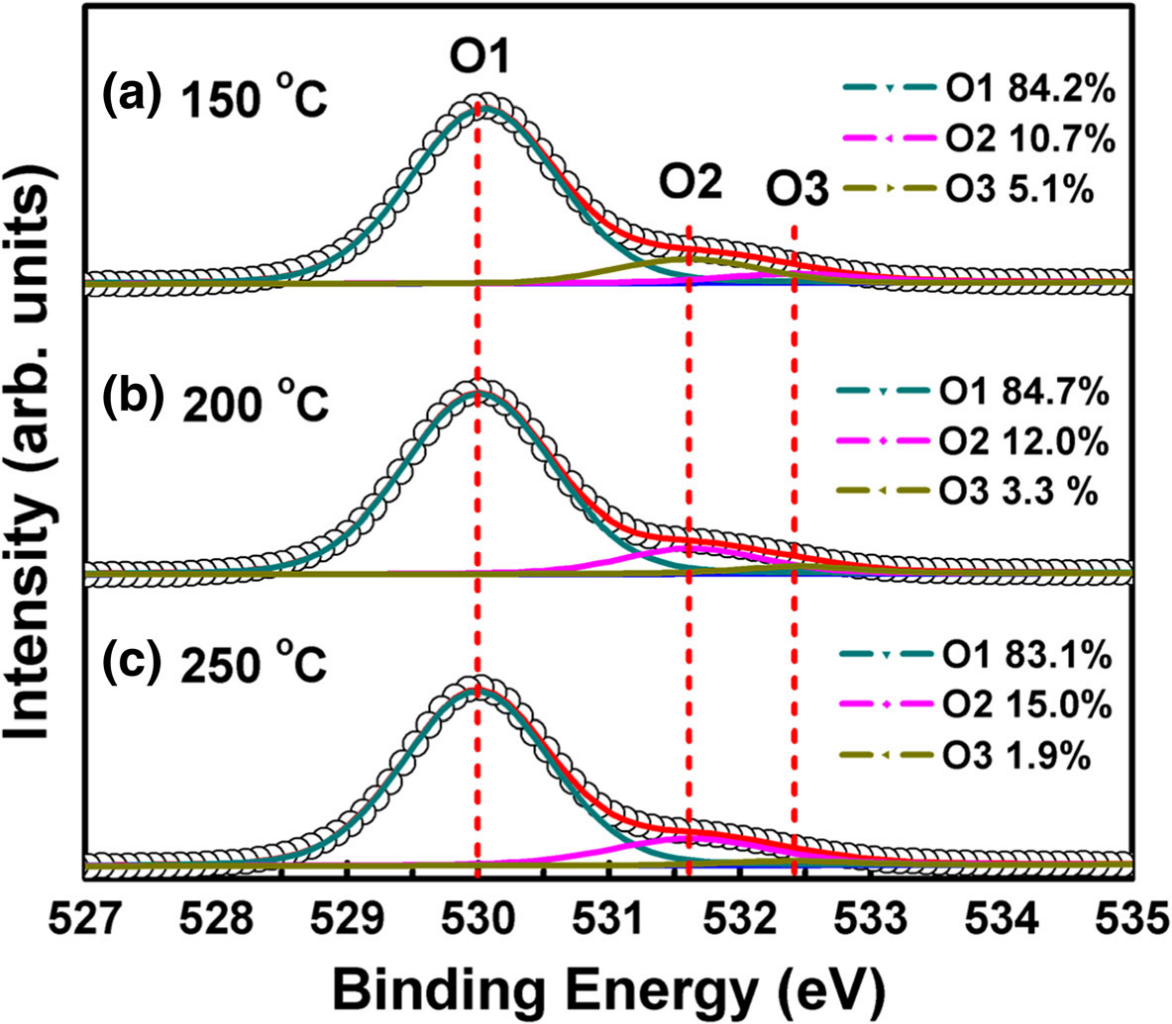
High-resolution O 1 s XPS spectra of the ZnO films grown at **a** 150 °C, **b** 200 °C, and **c** 250 °C, respectively

To further examine the effect of the growth temperature on the band alignment between ZnO and β-Ga_2_O_3_, the ZnO films are also grown at 150 and 250 °C. Note that ZnO films prepared by ALD at the temperatures of 150–250 °C have poly-crystalline nature. Figure 4 shows the high-resolution O 1s XPS spectra of the ZnO films grown at different temperatures. Each O 1s spectrum can be well separated into three components using Gaussian-Lorentzian function. The peaks centered at 530.0 (O1), 531.6 (O2), and 532.4 (O3) eV correspond to the Zn-O bands, oxygen vacancies, and –OH group [28, 29], respectively. The relative percentage of different components is also calculated according to the peak area, digested in Fig. 4. It shows that the relative content of oxygen vacancies increases from 10.7 to 15.0% due to the decomposition of precursors and the increase of Zn interstitials. However, the –OH counterpart reduces from 5.1 to 1.9% because of more complete reactions between DEZ precursors and surface –OH groups in this temperature range [30].

**Fig. 5:**
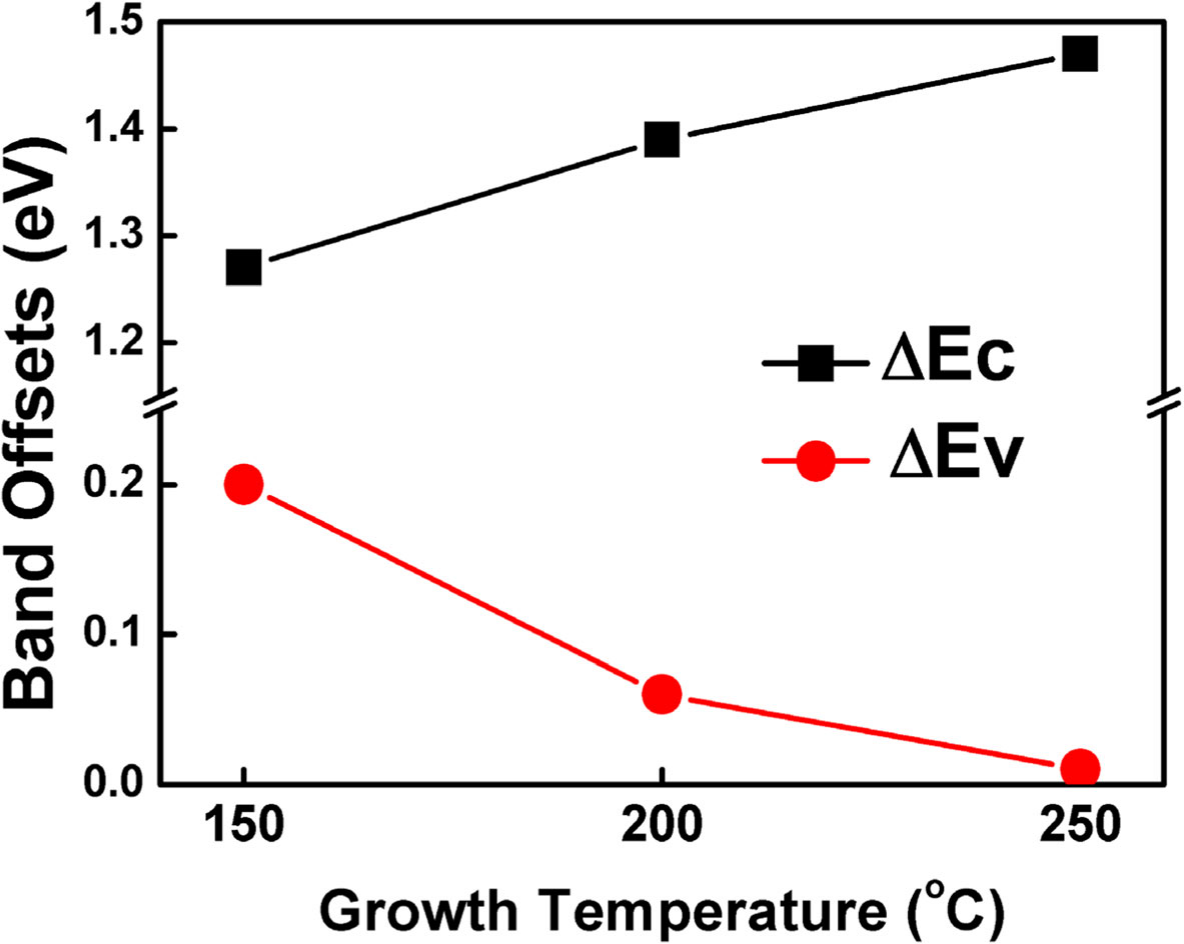
The conduction and valence band offsets of atomic-layer-deposited ZnO/β-Ga_2_O_3_ heterojunctions fabricated at different temperatures

Figure 5 shows the band offsets of ZnO/β-Ga_2_O_3_ heterojunctions as a function of growth temperature. The CBO increases from 1.26 to 1.47 eV with the growth temperature varying from 150 to 250 °C. The native donor defects include the Zn anti-position, oxygen vacancies, and Zn interstitials. However, the formation energy of anti-position atoms is so high that its concentration is extremely low. The Zn interstitials have more influence on the conduction band minimum (CBM) than that of the oxygen vacancy because the CBM is mainly dominated by the 4s orbit of Zn atom. [31] As a result, the increased CBO of 0.21 eV could be mainly contributed by Zn interstitials. On the other hand, the VBO reduces from 0.20 to 0.01 eV with the growth temperature increasing from 150 to 250 °C. The native acceptor defects include the O anti-position, Zn vacancies, and oxygen interstitials [32], whose formation energies are high and their number can be even negligible. Furthermore, the most native acceptor levels are deep within the ZnO bandgap, thus they have little effect on the VBM [33]. However, V_zn_ + OH is favorable to be presented duo to the low formation energy, [34] V_zn_ + OH may occur with an electron belonging to OH bonds. The lattice hydrogen H^+^ ion acts as a compensating center, and it can bind with the V_Zn_ around the dislocation and stacking faults core, ensuring the acceptor-type complex defect for p-type conductivity [35]. More residual –OH groups in the ZnO film are obtained at a lower growth temperature, i.e., 150 °C [36]. The acceptor level near the VBM reduces with the temperature, leading to an effectively downward shift in *E*_*V*_ of ZnO, thus the *∆E*_*V*_ becomes lower. Therefore, the ZnO deposited at lower temperature could be more efficiently to reduce the barrier height at the interface between the metal and Ga_2_O_3_.

## Conclusions

In summary, the energy band alignment at atomic-layer-deposited ZnO/β-Ga_2_O_3_ ($\overline{2}01$) was characterized by XPS. A type-I band alignment formed at the ZnO/β-Ga_2_O_3_ interface. The conduction band offset increased from 1.26 to 1.47 eV while the valence band offset decreased from 0.20 to 0.01 eV with the temperature increasing from 150 to 250 °C. These observations suggest that the ZnO deposited at lower temperature is favorable to be a promising ISL to reduce the electron barrier height at the ZnO/β-Ga_2_O_3_ interface.

## Abbreviations

ALD: Atomic layer deposition
CBM: Conduction band minimum
CBO: Conduction band offset.
CVD: Chemical vapor deposition
DEZ: Zn (C_2_H_5_)_2_
Ga_2_O_3_: Gallium oxide
GaN: Gallium nitride
ISL: Intermediate semiconductor layer
MOSFETs: Metal-oxide-semiconductor field-effect transistors
OH: Hydroxyl
SiC: Silicon carbide
UV-VIS: Ultraviolet-visible spectroscopy
VBM: Valence band maximum
VBO: Valence band offset
XPS: X-ray spectroscopy
ZnO: Zinc oxide

## References

[1] Rafique S, Han L, Zhao H (2017). Ultrawide bandgap β-Ga_2_O_3_ thin films: growths, properties and devices. ECS Trans.

[2] Si M, Yang L, Zhou H, Ye PD (2017) β-Ga2O3 nanomembrane negative capacitance field-effect transistors with steep subthreshold slope for wide band gap logic applications. ACS Omege 2:7136–7140 10.1021/acsomega.7b01289 PMC6645059 31457293

[3] Mastro MA, Kuramata A, Calkins J, Kim J, Ren F, Peartong SJ (2017). Opportunities and future directions for Ga_2_O_3_. ECS J Solid State Sc.

[4] Green AJ, Chabak KD, Heller ER, Fitch RC, Baldini M, Fiedler A, Irmscher K, Wagner G, Galazka Z, Tetlak SE, Crespo A, Leedy K, Jessen GH (2016). 3.8-MV/cm breakdown strength of MOVPE-grown Sn-doped *β*-Ga_2_O_3_ MOSFETs. IEEE Electron Device Lett.

[5] Moser NA, McCandless JP, Crespo A, Leedy KD, Green AJ, Heller ER, Chabak KD, Peixoto N, Jessen GH (2017). High pulsed current density β-Ga_2_O_3_ MOSFETs verified by an analytical model corrected for interface charge. Appl Phys Lett.

[6] Baldini M, Albrecht M, Fiedler A, Irmscher K, Klimm D, Schewski R, Wagner G (2016). Semiconducting Sn-doped β-Ga_2_O_3_ homoepitaxial layers grown by metal organic vapour-phase epitaxy. J Mater Sci.

[7] Feng W, Wang X, Zhang J, Wang L, Zheng W, Hu PA, Cao W, Yang B (2014). Synthesis of two-dimensional β-Ga_2_O_3_ nanosheets for high-performance solar blind photodetectors. J Mater Chem C.

[8] Higashiwaki M, Sasaki K, Kamimura T, Wong MH, Krishnamurthy D, Kuramata A, Masui T, Yamakoshi S (2013). Depletion-mode Ga_2_O_3_ metal-oxide-semiconductor field-effect transistors on β-Ga_2_O_3_ (010) substrates and temperature dependence of their device characteristics. Appl Phys Lett.

[9] Higashiwaki M, Sasaki K, Kuramata A, Masui T, Yamakoshi S (2012). Gallium oxide (Ga_2_O_3_) metal-semiconductor field-effect transistors on single-crystal β-Ga_2_O_3_ (010) substrates. Appl Phys Lett.

[10] Carey PH, Yang J, Ren F, Hays DC, Pearton SJ, Jang S, Kuramata A, Kravchenko II (2017). Ohmic contacts on n-type β-Ga_2_O_3_ using AZO/Ti/au. AIP Adv.

[11] Wu Z, Jiao L, Wang X, Guo D, Li W, Li L, Huang F, Tang W (2017). A self-powered deep-ultraviolet photodetector based on an epitaxial Ga_2_O_3_/Ga:ZnO heterojunction. J Mater Chem C.

[12] Huan YW, Sun SM, Gu CJ, Liu WJ, Ding SJ, Yu HY, Xia CT, Zhang DW (2018). Recent advances in β-Ga_2_O_3_-metal contacts. Nanoscale Res Lett.

[13] Hong SK, Hanada T, Makino H, Chen Y, Ko HJ, Yao T (2001). Band alignment at a ZnO/GaN (0001) heterointerface. Appl Phys Lett.

[14] Shi K, Zhang PF, Wei HY, Jiao CM, Li CM, Liu XL, Yang SY, Zhu QS, Wang ZG (2012). Energy band alignment of MgO (111)/ZnO (0002) heterojunction determined by X-ray photoelectron spectroscopy. Solid State Commun.

[15] Zhao B, Wang F, Chen H, Zheng L, Su L, Zhao D, Fang X (2017). An ultrahigh responsivity (9.7 mA W^−1^) self-powered solar-blind photodetector based on individual ZnO–Ga_2_O_3_ Heterostructures. Adv Funct Mater.

[16] Guo DY, Shi HZ, Qian YP, Lv M, Li PG, Su YL, Liu Q, Chen K, Wang SL, Cui C (2017). Fabrication of β-Ga_2_O_3_/ZnO heterojunction for solar-blind deep ultraviolet photodetection. Semicond Sci Technol.

[17] Wei L, Liu QX, Zhu B, Liu WJ, Ding SJ, Lu HL, Jiang A, Zhang DW (2016). Low-cost and high-productivity three-dimensional nanocapacitors based on stand-up ZnO nanowires for energy storage. Nanoscale Res Lett.

[18] Ohnishi S, Hirokawa Y, Shiosaki T, Kawabata A (1978). As-grown CVD ZnO optical waveguides on sapphire. Appl Phys Lett.

[19] Shen K, Wu K, Wang D (2013). Band alignment of ultra-thin hetero-structure ZnO/TiO_2_ junction. Mater Res Bull.

[20] Chen M, Zhao B, Hu G, Fang X, Wang H, Wang L, Luo J, Han X, Wang X, Pan C, Wang ZL (2018). Piezo-phototronic effect modulated deep UV photodetector based on ZnO-Ga_2_O_3_ Heterojuction microwire. Adv Funct Mater.

[21] Eom K, Lee D, Kim S, Seo H (2018). Modified band alignment effect in ZnO/Cu_2_O heterojunction solar cells via Cs_2_O buffer insertion. J Phys D Appl Phys.

[22] Jia Y, Zeng K, Wallace JS, Gardella JA, Singisetti U (2015) Spectroscopic and electrical calculation of band alignment between atomic layer deposited SiO_2_ and β-Ga_2_O_3_ ($$ \overline{2}01 $$). Appl Phys Lett 106:102107

[23] Dewan S, Tomar M, Goyal A, Kapoor AK, Tandon RP, Gupta V (2016). Study of energy band discontinuity in NiZnO/ZnO heterostructure using X-ray photoelectron spectroscopy. Appl Phys Lett.

[24] Kraut EA, Grant RW, Waldrop JR, Kowalczyk SP (1980) Precise determination of the valence-band edge in X-ray photoemission spectra: application to measurement of semiconductor interface potentials. Phys Rev Lett 44(24):1620–1623

[25] You JB, Zhang XW, Song HP, Ying J, Guo Y, Yang AL, Yin ZG, Chen NF, Zhu QS (2009). Energy band alignment of interface determined by x-ray photoelectron spectroscopy. J Appl Phys.

[26] Carey PH, Ren F, Hays DC, Gila BP, Pearton SJ, Jang S, Kuramata A (2017). Valence and conduction band offsets in AZO/Ga_2_O_3_ heterostructures. Vacuum.

[27] Carey PH, Ren F, Hays DC, Gila BP, Pearton SJ, Jang S, Kuramata A (2017) Band alignment of atomic layer deposited SiO_2_ and HfSiO_4_ with ($$ \overline{2}01 $$) β-Ga_2_O_3_. Jpn J Appl Phys 56:071101

[28] Major S, Kumar S, Bhatnagar M, Chopra KL (1986). Effect of hydrogen plasma treatment on transparent conducting oxides. Appl Phys Lett.

[29] Wang ZG, Zu XT, Zhu S, Wang LM (2006). Green luminescence originates from surface defects in ZnO nanoparticles. Phys E.

[30] Saha D, Das AK, Ajimsha RS, Misra P, Kukreja LM (2013). Effect of disorder on carrier transport in ZnO thin films grown by atomic layer deposition at different temperatures. J Appl Phys.

[31] Kobayashi A, Sankey OF, Volz SM, Dow JD (1983). Semiempirical tight-binding band structures of wurtzite semiconductors: AlN, CdS, CdSe, ZnS, and ZnO. Phys Rev B.

[32] Heiland G, Mollwo E, Stockmann Z (1959). Electronic processes in zinc oxide. Solid State Phys.

[33] Vidya R, Ravindran P, Fjellvag H, Svensson BG, Monakhov E, Ganchenkova M, Nieminen RM (2011). Energetics of intrinsic defects and their complexes in ZnO investigated by density functional calculations. Phys Rev B.

[34] Xue X, Liu L, Wang Z, Wu Y (2014). Room-temperature ferromagnetism in hydrogenated ZnO nanoparticles. J Appl Phys.

[35] Senthilkumar K, Yoshida T, Fujita Y (2018). Formation of D–V Zn complex defects and possible p-type conductivity of ZnO nanoparticle via hydrogen adsorption. J Mater Sci.

[36] Fang M, Qi L, Zhang C, Chen Q (2016). Effects of thickness and deposition temperature of ALD ZnO on the performance of inverted polymer solar cells. J Mater Sci Mater Electron.

